# Chronic Human Pegivirus 2 without Hepatitis C Virus Co-infection

**DOI:** 10.3201/eid2602.190434

**Published:** 2020-02

**Authors:** Kelly E. Coller, Veronica Bruce, Michael Cassidy, Jeffrey Gersch, Matthew B. Frankel, Ana Vallari, Gavin Cloherty, John Hackett, Jennifer L. Evans, Kimberly Page, George J. Dawson

**Affiliations:** Abbott Laboratories, Abbott Park, Illinois, USA (K.E. Coller, M. Cassidy, J. Gersch, M.B. Frankel, A. Vallari, G. Cloherty, J. Hackett, Jr., G.J. Dawson);; University of New Mexico, Albuquerque, New Mexico, USA (V. Bruce, K. Page);; University of California San Francisco, San Francisco, California, USA (J.L. Evans)

**Keywords:** bloodborne pathogens, prevalence, substance abuse, intravenous drug use, hepatitis virus, hepatitis C, serologic test, viruses, HPgV-2, human pegivirus 2, co-infection, pegivirus

## Abstract

Most human pegivirus 2 (HPgV-2) infections are associated with past or current hepatitis C virus (HCV) infection. HPgV-2 is thought to be a bloodborne virus: higher prevalence of active infection has been found in populations with a history of parenteral exposure to viruses. We evaluated longitudinally collected blood samples obtained from injection drug users (IDUs) for active and resolved HPgV-2 infections using a combination of HPgV-2–specific molecular and serologic tests. We found evidence of HPgV-2 infection in 11.2% (22/197) of past or current HCV-infected IDUs, compared with 1.9% (4/205) of an HCV-negative IDU population. Testing of available longitudinal blood samples from HPgV-2–positive participants identified 5 with chronic infection (>6 months viremia in >3 timepoints); 2 were identified among the HCV-positive IDUs and 3 among the HCV-negative IDUs. Our findings indicate that HPgV-2 can establish chronic infection and replicate in the absence of HCV.

The recently identified second human pegivirus (HPgV-2 or HHpgV-1) is a bloodborne flavivirus: little is known about the potential clinical significance of infection (*1*,*2*). Active or resolved HPgV-2 infection has been detected worldwide in cohorts associated with risk for parenteral exposure to bloodborne pathogens (*1*,*3*–*5*). In a study in which hepatitis C virus (HCV) status was determined (*3*), 1.2% of HCV positive were actively infected with HPgV-2; none of the 1,306 HCV-negative participants (volunteer blood donors, HBV infected, HIV infected) were actively infected. In another study, participants with concurrent HIV/HCV infection or injection drug users (IDUs) had a higher prevalence (3.0%–5.7%) of active HPgV-2 infection (*4*–*6*). Although active HPgV-2 has been found in other populations (e.g., hemophiliacs or others, with risk for parenteral exposure), their HCV antibody status was not determined (*6*,*7*).

Previous studies indicate that HPgV-2 can establish a chronic infection characterized by detectable viremia for >6 months (*2*,*6*). Most chronic HPgV-2 cases are associated with active HCV infection (*2*,*6*). In chronic HPgV-2 cases in which HCV RNA has not been detected, the presence of HCV antibodies (indicating a resolved infection) was not determined; thus, it is unclear whether previous HCV infection played a role in the initial HPgV-2 infection. Despite observations of HCV and HPgV-2 co-infection, no evidence has been reported that HPgV-2 infection exacerbates HCV infection (*5*,*6*,*8*) or that co-infection influences the replication of either virus.

We examined a cohort of IDUs for whom longitudinal samples were available. We monitored the cohort for HCV status by HCV antibodies, RNA, or both, with the intent of capturing nascent HCV infections (*9*). We performed initial testing for HPgV-2 (RNA and antibodies) on baseline and last collected samples. We further characterized longitudinal samples from available participants that showed active or resolved HPgV-2 infection upon testing initial or last timepoints. We hypothesized that the IDUs would have similar prevalence of HPgV-2 as shown in a previous study of HCV-infected persons with unknown IDU status, and that by studying IDUs without HCV infection we would uncover HPgV-2 infection in the absence of HCV. Last, we hypothesized that longitudinal samples from IDUs would reveal whether HPgV-2 can establish a persistent infection in the absence of HCV co-infection.

## Materials and Methods

### Samples

We obtained samples from the U-Find-Out (UFO) Study, an ongoing prospective observational study of young adult active injectors, <30 years of age at enrollment, that was initiated in 2003 in the San Francisco Bay area (California, USA). Details of enrollment methods and follow-up have been described previously (*9*,*10*). In brief, young adult IDUs were recruited from neighborhoods where IDUs were known to congregate and invited to participate in a field study for eligibility screening. Eligible persons were those who reported injection drug use in the prior 30 days, were <30 years of age, spoke English, had no plans to travel outside of the San Francisco Bay area for >3 months, and had negative or unknown HCV status. HCV antibody–positive persons were admitted into the study if their HCV RNA status was negative or unknown; those identified as HCV infected (RNA positive) at baseline were not enrolled into follow-up. 

Eligible consenting participants were asked to complete a baseline interviewer-administered structured questionnaire that queried sociodemographics, parenteral and sexual risk behaviors and exposures, injecting exposures (e.g., frequency of injecting, number of persons injected with, types of drugs injected), alcohol use, and prevention and health service use. They were also asked to provide blood samples for HCV testing, including both HCV antibodies and HCV RNA, and for storage. Before 2012, all participants provided samples for HCV antibodies (using standard laboratory-based testing) and for a qualitative HCV RNA status determination using a nucleic acid amplification test (Procliex HIV-1/HCV assay; Gen-Probe Inc., https://www.novartis.com). Beginning in May 2012, HCV antibody testing was primarily conducted using a rapid test (OraSure Technologies, https://www.orasure.com) by fingerstick capillary blood collection; however, venipuncture was still used to collect specimens for RNA testing and sample storage. Baseline samples on 402 participants were selected as the initial sample set (Figure). A total of 205 (51.0%) samples were negative and 197 (49.0%) positive for HCV antibodies and HCV RNA at baseline. HCV-positive in this current study is defined as any evidence of HCV infection (RNA or HCV antibodies), past or present; HCV-negative is defined as no evidence (RNA or HCV antibodies), past or present.

**Figure:**
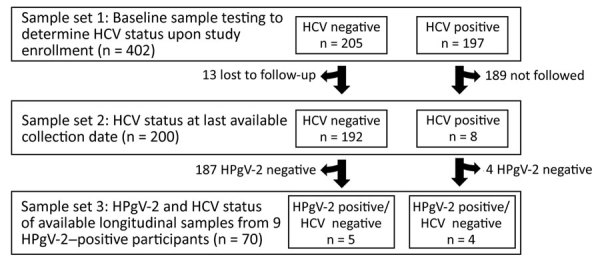
Design of study of chronic human pegivirus-2 and hepatitis C virus co-infection in injection drug users in the San Francisco Bay area, California, USA. Samples were tested using HPgV-2 molecular and serologic assays in 3 sample sets. HCV, hepatitis C virus; HPgV-2, human pegivirus 2.

### HPgV-2 Prevalence Study Design

We used previously described HPgV-2 molecular (*11*) and serologic (*3*) assays to test all samples for determining HPgV-2 prevalence. We divided them into 3 testing groups: sample set 1, initial blood samples (n = 402); sample set 2, all last available follow-up samples (n = 200); and sample set 3, any longitudinal samples available for samples that were HPgV-2 (RNA or antibody) positive at initial or last draw timepoint (n = 70) (Figure). Because the initial study collection targeted incident HCV infection, a limited number of participants who were HCV positive at initial collection had follow-up samples available; only 8 HCV-positive and 192 HCV-negative participants from sample set 1 had follow-up (last) draw available for testing, constituting sample set 2.

### HPgV-2 Molecular Assay

We used a modified version of the HPgV-2 reverse transcription PCR (RT-PCR) to determine HPgV-2 viremia (*11*). The RT-PCR targets 2 conserved regions of the HPgV-2 genome within the 5′ untranslated region (UTR) and the nonstructural (NS) 2–3 coding region (*11*). We modified the assay to replace detection of HPgV RNA with an internal control. The internal control was derived from the hydroxypyruvate reductase gene from the pumpkin plant, *Cucurbita pepo*, and is delivered in an Armored RNA (Ambion, Inc., https://www.thermofisher.com) particle that has been diluted in negative human plasma (nonreactive for HBsAg, HIV RNA, HCV RNA, HBV DNA, HIV-1/-2 antibodies, and HCV antibodies). We introduced the internal control into each specimen at the beginning of sample preparation as a control for extraction and amplification. We extracted samples from plasma using the Abbott *m*2000*sp* instrument (Abbott Molecular, https://www.molecular.abbott) (open mode protocol m2000-RNA-Plasma-LL-500–110-v71408, version 1.0). We used eluted nucleic acids immediately for subsequent PCR analysis or stored them in the deep well plate at −80°C.

### HPgV-2 Antibody Testing

We screened research use–only assays to detect IgG response to HPgV-2 proteins for HPgV-2 seroconversion (*3*). In brief, we built 2 separate indirect IgG assays for use on the ARCHITECT instrument (Abbott Laboratories). The capture antigen for the E2 assay was mammalian expressed glycoprotein E2, and for the NS4AB assay a portion of the NS4AB region. We generated signal to cutoff values for each assay by determining a provisional cutoff from testing a population of low-risk volunteer donors and calculating the median + 10 SD of relative light units (RLU) generated using the individual assays (*3*). Both E2 and NS4AB assays detected active and resolved HPgV-2 infection (*3*).

### Statistical Analyses

We used the Fisher exact test to examine differences in prevalence of HPgV-2 between subgroups (for example, by HCV status). We performed unpaired Student *t*-tests to determine if there was a significant difference (p<0.05) between the average HPgV-2 log copies/mL (NS2/3 or 5'UTR) of the HCV positive and negative groups. We used GraphPad Prism version 6.04 for Windows (GraphPad Software, https://www.graphpad.com).

## Results

### Baseline Sample Testing

The overall prevalence of HPgV-2 (presence of RNA or antibodies) among baseline samples in the IDU cohort was 6.5% (Table 1; Figure). We determined a higher HPgV-2 prevalence in the HCV-positive group (11.2%) compared with the HCV-negative group (1.9%) (p = 0.0002 by Fisher exact test). We observed HPgV-2 infection (HPgV-2 RNA) more frequently in the HCV-positive group (6.1%; 12/197 samples) than in the HCV-negative group (1.0%; 2/197 samples).

**Table 1:** Prevalence of HPgV-2 antibodies or RNA, initial blood draw samples from study of chronic HPgV-2 infection and HCV co-infection in injection drug users in the San Francisco Bay area, California, USA*

HCV status	No. tested	No. HPgV-2 Ab+	No. HPgV-2 Ab+/RNA+	No. HPgV-2 Ab−/RNA+	Total RNA+	Total HPgV-2 RNA or Ab+ (%)
HCV positive	197	18	8	4	12	22 (11.2)
HCV negative	205	3	1	1	2	4 (1.9)
Totals	402	21	9	5	14	26 (6.5)

*Ab, antibody; HCV, hepatitis C virus; HPgV-2, human pegivirus 2; +, positive; −, negative

### Last Sample Testing

Follow-up specimens were available for some study participants (sample set 2), primarily those who were HCV negative, due to the prospective design of the study that did not require follow-up samples from HCV-positive participants (Figure). However, the study also included persons with newly detected HCV infection whom we followed to examine natural history and resolution of incident HCV infection (*9*). A total of 200 participants from baseline collection had a final follow-up sample available for evaluation in the HPgV-2 RNA and antibody assays; this group included 8 HCV-positive and 192 HCV-negative participants (Figure). Although 26 participants were HPgV-2 positive (by RNA or antibodies) at baseline (Table 1), only 4 participants were available for follow-up; they provided 3 HPgV-2 RNA-positive samples and 1 HPgV-2 RNA-negative seropositive sample.

We saw evidence of HPgV-2 infection (antibodies or RNA) in 9 samples in set 2, 6 HCV-negative samples and 3 HCV-positive samples (Table 2). Among the HCV-negative participants, 3 (QM0003, VH0052, VP0295) showed active HPgV-2 infection during either the baseline or last draw timepoints. Participant QM0003 showed chronic (>6 mos viremia) HPgV-2 infection during the study, with detection of HPgV-2 RNA at time points spanning 832 days (Table 2). Participant VH0052 was actively infected with HPgV-2 at baseline and resolved infection by the last draw date (201 days elapsed), whereas participant VP0295 acquired HPgV-2 infection during the study and was RNA positive at the last draw date (on study for 553 days). Three participants (GG0012, RM0095, RM0337) were HPgV-2 RNA and antibody negative at enrollment and had seroconverted to have HPgV-2 antibodies by the last draw. One participant, VH0044, was HPgV-2 seropositive at baseline but was seronegative (seroreverted) by the final draw (250 days elapsed).

**Table 2:** Evidence of HPgV-2 infection in most recent samples from study of chronic HPgV-2 infection and HCV co-infection in injection drug users in the San Francisco Bay area, California, USA*

HCV RNA status at initial draw	Sample ID	HPgV-2 status at initial blood draw			HPgV-2 status at last blood draw		HCV RNA status	No. days†	Comments‡
		RNA	Antibody		RNA	Antibody			
Negative	QM0003	Pos	Neg		Pos	Pos	Neg	832	Chronic
	VH0052	Pos	Pos		Neg	Pos	Neg	201	Resolved
	VP0295	Neg	Neg		Pos	Pos	Neg	553	Active
	GG0012	Neg	Neg		Neg	Pos	Neg	689	Resolved
	RM0095	Neg	Neg		Neg	Pos	Neg	201	Resolved
	RM0337§	Neg	Neg		Neg	Pos	Pos	1,680	Resolved
	VH0044	Neg	Pos		Neg	Neg	Neg	250	Resolved
Positive	VH0085	Pos	Pos		Pos	Pos	Neg	2,805	Chronic
	GG0038	Neg	Neg		Pos	Neg	Neg	461	Active
	VT0031	Neg	Neg		Pos	Pos	Pos	818	Active

*HCV, hepatitis C virus; HPgV-2, human pegivirus 2; neg, negative; pos, positive.  †Days elapsed between initial and last blood draw. ‡Comments of HPgV-2 status based on initial and last draw testing. Chronic indicates >6 months with detectable active viremia. Active indicates viremia at last draw. Resolved indicates no viremia detected at last draw, but antibodies were present. §Participant RM0337 acquired HCV during the study.

In the HCV-positive group, 1 participant (VH0085) was HPgV-2 RNA positive both on the first and last draw dates (2,805 days elapsed). The other 2 HCV-positive participants (GG0038 and VT0031) were HPgV-2 RNA negative on the first draw dates but showed active HPgV-2 infection at the last draw date. A single participant (RM0337) acquired both HPgV-2 and HCV during the course of the study, with HPgV-2 infection preceding HCV infection by 280 days (Table 3). Within the last sample set, 6 participants demonstrated HPgV-2 infection after initial collection. Three of the participants showed active HPgV-2 viremia and 3 showed resolved HPgV-2 infection as indicated by detection of antibodies only.

**Table 3:** Information about participants with HPgV-2 infection with HCV co-infection in study of injection drug users in the San Francisco Bay area, California, USA*

Sample ID	Participant age, y/sex	No. years drug use†	Collection date	HCV RNA	HCV antibody	NS2/3 log_10_ copies/mL	5′ UTR log_10_ copies/mL	NS4AB S/CO‡	E2 S/CO‡
RM0337	25.6/F	11.1	2013 Jul 10	Neg	Neg	Neg	Neg	0.16	0.14
			2013 Oct 9	Neg	Neg	Neg	Neg	0.14	0.13
			2014 Jan 29	Neg	Neg	Neg	Neg	0.13	0.12
			2016 Jul 27	Neg	Neg	Neg	Neg	0.12	0.11
			2016 Oct 26	Neg	Neg	Neg	Neg	0.12	0.13
			2017 Jan 18	Neg	Neg	Neg	Neg	0.13	0.13
			2017 Apr 26	Neg	Neg	0.96	0.42	0.13	1.46
			2017 Jul 19	Neg	Neg	Neg	Neg	0.09	1.34
			2017 Oct 18	Neg	Neg	Neg	Neg	0.13	0.88
			2018 Jan 31	Pos	Neg	Neg	Neg	0.14	1.02
			2018 Feb 14	Pos	Neg	Neg	Neg	0.25	1.26
VH0085§	22.2/F	9.9	2010 May 14	Pos	Pos	3.22	2.3	1.38	9.37
			2017 Feb 15	Neg	Not tested	3.14	2.07	2.96	12.92
			2017 May 17	Neg	Not tested	1.87	0.69	3.33	12.35
			2017 Aug 9	Neg	Not tested	4.30	2.25	6.13	18.71
			2017 Nov 1	Neg	Not tested	3.40	1.68	5.13	19.45
			2018 Jan 17	Neg	Not tested	3.86	2.26	3.75	16.16
GG0038§	26.5/M	8.8	2016 Jul 14	Pos	Pos	Neg	Neg	0.16	0.12
			2017 Mar 1	Pos	Not tested	3.04	1.02	0.17	¶
			2017 May 3	Pos	Not tested	2.14	0.64	0.20	0.37
			2017 May 23	Neg	Not tested	3.01	1.16	0.18	0.36
			2017 Oct 18	Neg	Not tested	4.15	1.91	0.16	2.96
VT0031	19.8/M	1.8	2005 Feb 2	Pos	Pos	Neg	Neg	0.09	0.22
			2005 May 24	Pos	Pos	Neg	Neg	0.11	0.15
			2005 Jun 14	Pos	Pos	Neg	Neg	0.10	0.17
			2005 Aug 9	Pos	Pos	Neg	Neg	0.12	0.14
			2005 Nov 8	Pos	Pos	Neg	Neg	0.08	0.13
			2006 Jan 31	Pos	Pos	Neg	Neg	0.14	0.13
			2006 May 9	Pos	Pos	Neg	Neg	0.09	0.19
			2006 Aug 8	Pos	Pos	Neg	Neg	0.21	1.25
			2007 Feb 6	Pos	Pos	2.30	0.53	0.14	1.19
			2007 May 1	Pos	Pos	0.48	0.41	0.14	1.82

*Participants were determined positive for HCV by RNA or antibody test. HCV, hepatitis C virus; HPgV-2, human pegivirus 2; ID, identification; neg, negative; NS, nonstructural protein; pos, positive; S/CO, signal to cutoff value; UTR, untranslated region.  †Number of years participant had injected drugs as of the time of the initial blood draw.  ‡S/CO >1.0 is considered positive. §Participants VH0085 and GG0038 demonstrate resolved HCV infection. VH0085 was administered direct active antiviral drugs (8 weeks ledipasvir/sofosbuvir, LDV-SOF), and GG0038 spontaneously cleared HCV infection. ¶Volume not available for testing.

### Longitudinal Sample Testing

We tested longitudinal samples (N = 70, sample set 3) from 9/10 participants (Table 2) for HPgV-2 antibodies and RNA (Tables 3, 4; Figure). We reported data available for age, sex, years of injection drug use, HIV status, HCV RNA, HCV antibodies, alanine aminotransferase (HCV-positive only), and HCV drug treatment therapy. We did not follow up with additional HPgV-2 testing on participant VH0044, who was negative for HPgV-2 RNA and antibodies (seroreverted) by last sample date (Table 2). We found chronic HPgV-2 (active viremia >6 months) in 5 different IDU participants, 3 male and 2 female; 3 (QM0003, VP0295, RM0095) were HCV negative and 2 (VH0085, GG0038) HCV positive. None of the participants demonstrating chronic HPgV-2 infection or seroconversion had evidence of HIV infection. Among the participants who demonstrated chronic HPgV-2 infection and were HCV negative, participant QM0003 had a longer (>5 y) history of exposure to injection drug use than the other participants, RM0095 and VP0295 (<5 y). Both participants with active HPgV-2 and HCV co-infection (GG0038 and VH0085) had long histories of injecting exposure (>5 y) and maintained HPgV-2 after HCV infection was resolved.

**Table 4:** Information about participants with HPgV-2 infection without HCV co-infection in study of injection drug users in the San Francisco Bay area, California, USA*

Sample ID	Age, y/sex	No. years drug use†	Collection date	NS2/3 log_10 _copies/mL	5′ UTR log_10 _copies/mL	NS4AB S/CO‡	E2 S/CO‡
QM0003	28.4/F	14.4	2015 Oct 10	3.76	2.84	0.12	0.08
			2015 Nov 4	0.93	0.55	0.08	0.08
			2016 Jan 27	2.66	1.10	0.16	0.72
			2016 Apr 18	3.56	1.62	0.07	0.85
			2016 Jul 13	3.66	1.61	§	§
			2016 Oct 5	3.22	1.74	0.13	2.76
			2017 Jan 4	4.22	2.30	0.25	4.67
			2017 Apr 10	3.85	2.10	0.17	3.58
			2017 Jul 5	4.08	2.39	0.28	3.73
			2018 Feb 7	3.41	1.34	0.37	2.66
VP0295	24.6/M	3.1	2016 Jan 6	Neg	Neg	0.10	0.12
			2016 Apr 6	Neg	Neg	0.09	0.11
			2016 Aug 24	Neg	Neg	0.12	0.11
			2017 Jan 4	4.44	2.82	0.11	0.14
			2017 Apr 12	3.19	1.06	0.09	0.89
			2017 Jul 12	1.42	0.82	0.15	5.51
GG0012	23.4/F	3.4	2016 Apr 29	Neg	Neg	0.23	0.52
			2016 Aug 17	Neg	Neg	1.77	3.28
			2016 Nov 29	Neg	Neg	3.18	5.76
			2017 Mar 1	Neg	Neg	4.90	8.90
			2017 May 31	Neg	Neg	2.60	4.51
			2017 Sep 20	Neg	Neg	4.05	10.94
			2017 Dec 13	Neg	Neg	6.03	11.04
			2018 Mar 19	Neg	Neg	3.77	6.67
VH0052	23.7/M	3.5	2006 Dec 14	2.13	1.23	2.00	1.91
			2007 Jul 3	Neg	Neg	0.81	1.63
RM0095	27.1/M	1.1	2011 Jan 19	Neg	Neg	0.06	0.06
			2011 Jul 27	0.02	0.14	0.07	0.09
			2011 Oct 19	2.33	1.54	0.06	0.29
			2012 Jan 11	0.20	0.50	0.09	0.59
			2012 Apr 11	0.02	0.16	0.24	0.50
			2012 Jul 3	Neg	Neg	0.14	0.59
			2012 Oct 23	0.03	0.27	0.12	1.09
			2013 Jan 15	0.45	0.61	0.15	2.45
			2013 Apr 10	1.37	1.08	0.13	2.37
			2013 Jul 2	Neg	Neg	0.17	3.53
			2013 Sep 18	0.46	0.55	0.14	2.64
			2013 Dec 11	Neg	Neg	0.14	3.07

*Participants were determined negative for HCV by RNA or antibody test. HCV, hepatitis C virus; HPgV-2, human pegivirus 2; ID, identification; neg, negative; NS, nonstructural protein; pos, positive; S/CO, signal to cutoff value; UTR, untranslated region.  †Number of years participant had injected drugs as of the time of the initial blood draw.  ‡S/CO >1.0 is considered positive. §No blood sample available for testing.

All chronic HPgV-2 infections demonstrated active viremia despite the presence of HPgV-2 antibodies, with most participants generating an IgG response to the glycoprotein E2 (Tables 3, 4). E2 antibodies developed in all participants with chronic HPgV-2 samples and observed seroconversion (GG0038, QM0003, VP0295, and RM0095) before the other marker, NS4AB antibody (Tables 3, 4). One chronically infected sample, VH0085, contained antibodies to both E2 and NS4AB, but we did not observe the initial seroconversion. Compared with the other samples in this study, VH0085 had the highest signal to cutoff values for both E2 and NS4AB antibody assays, and the HPgV-2 RNA log copies/mL were higher than in most other samples (Table 3). Some participants (VP0295, RM0095, GG0038) demonstrated seroconversion after several months of initial HPgV-2 RNA detection; these participants were chronically infected with HPgV-2.

The median using the NS2/3 assay was 3.26 HPgV-2 log_10_ copies/mL for the HCV-negative group and 3.21 HPgV-2 log_10_ copies/mL for the HCV-positive group; using the 5′ UTR, results were 1.71 log_10_ copies/mL for the HCV-negative group and 1.60 log_10_ copies/mL for the HCV-positive group (Table 5). HCV co-infection did not appear to influence HPgV-2 viral load; the average value showed no significant difference between the HCV-positive and HCV-negative groups (NS2/3, p = 0.11; 5′ UTR, p = 0.36). One HCV-positive participant, VH0085, was positive for HPgV-2 and HCV RNA at baseline, received HCV treatment (8 weeks ledipasvir/sofosbuvir), and cleared HCV infection. After clearance of HCV, the participant remained HPgV-2 viremic and went on to establish a chronic HPgV-2 infection that lasted 8 years (2,805 days). Participant GG0038 acquired HPgV-2 infection after spontaneous resolution of HCV infection (RNA negative and HCV antibody positive) and maintained active HPgV-2 infection for >232 days.

**Table 5:** Characteristics of findings in study of chronic HPgV-2 infection for participants with and without HCV co-infection in injection drug users in the San Francisco Bay area, California, USA*

Finding	HCV negative, n = 3	HCV positive, n = 2	p value
Average HPgV-2, log_10_ copies/mL			
NS2/3	3.26	3.21	0.11
5′ UTR	1.71	1.60	0.36
Average years injection drug use†	5.5	7.9	NA
Average age at detection of HPgV-2 RNA, y†	25.6	26.1	NA

*HCV, hepatitis C virus; HPgV-2, human pegivirus 2; NS, nonstructural protein. †No p values available because of low number of samples.

## Conclusions

The recently identified human pegivirus HPgV-2 has yet to be linked with any disease in humans. Several groups have shown HPgV-2 infection associated with HCV co-infection (*1*,*3*–*6*). HPgV-2 is a bloodborne virus, and a higher HPgV-2 prevalence is observed among HCV-positive IDUs (*4*,*6*). We decoupled the behavior of injection drug use from HCV status by monitoring HCV-negative or HCV-positive IDUs for HPgV-2 infection (RNA and antibodies). We also observed the enrichment of HPgV-2 infection in HCV-positive IDUs (Table 1), as was reported previously (*4*,*6*). We found, through longitudinal surveillance of both HCV-negative participants, that HPgV-2 can establish infection and maintain chronic infection in the absence of HCV. We defined chronic infection as detectable HPgV-2 viremia for >6 months in >3 timepoints that was not associated with particular symptoms. Of 9 participants with evidence of HPgV-2 infection (by RNA or serology), 2 HCV-infected participants demonstrated chronic HPgV-2 infection (Table 3), and 3 participants demonstrated chronic HPgV-2 without evidence of past or present HCV infection (Table 4).

Several limitations can contribute to the underestimation of chronic HPgV-2 infection. We identified HPgV-2 in baseline samples from 12 HCV-positive participants; but because of the study design most HCV-positive participants were not followed through subsequent timepoints. Three of these participants did provide longitudinal samples; 2 participants demonstrated chronic HPgV-2 infection. A second limitation is that participants testing negative for HPgV-2 during the timepoints evaluated may become positive following the last timepoint sampled, if they continue the risk behavior of intravenous drug use. Alternatively, false-positive chronic infections could result from long lapses in sampling, in which the participant could become infected, clear the infection, then become reinfected with HPgV-2. The molecular and serologic assays cannot distinguish reinfection from chronic infection.

We observed HPgV-2 seroconversion in the longitudinal surveillance of 7 participants; the detectable IgG response occurred several months after initial HPgV-2 RNA detection in several of the chronically infected participants (Tables 3, 4). Similar to HCV infection, which demonstrates a seronegative viremic window period of 50–60 days (*12*–*14*), the detection of HPgV-2–specific antibodies lagged behind detectable HPgV-2 RNA (Tables 3, 4). In this study, all participants demonstrating chronic HPgV-2 viremia did so in the presence of antibodies to the glycoprotein E2, which suggests that E2 antibodies are not neutralizing. In contrast, active viremia and E2 IgG are rarely co-detected in persons with the closest human virus, human pegivirus-1 (HPgV, GBV-C); the presence of E2 antibodies in HPgV infection often indicates resolution of infection (*3*,*15*–*17*).

Our data indicate that chronic HPgV-2 infection among IDUs does not require active HCV to establish infection or maintain chronic infection. It is possible that participants with no detectable HCV antibodies were seroreverters from previous cleared HCV infections; however, this is unlikely in our study because the cohort of active IDUs had ongoing exposure to HCV, and because seroreversion in immunocompetent persons has been shown to occur after a long time (>7 years) (*18*), exceeding the observation period of this study. Furthermore, 2 participants showed spontaneous (GG0038) or therapeutic (VH0085) resolution of HCV infection but HPgV-2 chronic infection remained (Table 3); thus, there appeared to be no reliance on HCV to establish or maintain HPgV-2 infection. We observed no difference in HPgV-2 viral loads whether HCV was present or absent (Table 5). The relative ratio of resolved to active infections between the HCV-positive and HCV-negative cohorts was similar (Table 1).

As noted in this and other studies, high-risk populations that are exposed to parenterally transmitted viruses experience an increase in HPgV-2 prevalence (*4*,*6*,*7*). We observed similar higher incidence of both active and resolved HPgV-2 infection in the HCV-positive IDU cohort (Table 1). The HCV-positive and HCV-negative infected persons within the IDU cohort share many common behaviors with no discernable characteristics, except total number of years of injection drug use (average 7.6 y for HCV-positive users, 4.7 y for HCV-negative users). Similarly, the HCV-positive HPgV-2 carriers identified in this study demonstrated injection drug use behavior longer (average 7.9 y) than the HCV-negative group (average 4.4 y). The greater number of potential exposures to parentally transmitted bloodborne viruses is probably a major contributing factor for increased prevalence of HPgV-2 in the HCV-positive IDU cohort.

The pathogenic potential of HPgV-2 in humans remains unknown; no clinical symptoms have been associated with HPgV-2 infection. We gathered no additional clinical information from HCV-negative study participants. Most HPgV-2 studies have shown the virus is associated with HCV co-infection, which can mask any pathogenicity associated with HPgV-2 infection. Identifying populations that show higher prevalence of HPgV-2 monoinfection and monitoring these persons over time may help identify clinical symptoms associated with HPgV-2 infection, thus enabling researchers to categorize HPgV-2 as human pathogen or benign infection.
